# Endoscopic Extracapsular Removal of Pituitary Adenoma: The Importance of Pretreatment of an Adjacent Unruptured Internal Carotid Artery Aneurysm

**DOI:** 10.1155/2012/891847

**Published:** 2012-10-18

**Authors:** So Yamada, Shoko M. Yamada, Toshio Hirohata, Yudo Ishii, Katsumi Hoya, Mineko Murakami, Akira Matsuno

**Affiliations:** ^1^Department of Neurosurgery, Teikyo University Chiba Medical Center, Ichihara, Chiba 299-0111, Japan; ^2^Department of Neurosurgery, University of Tokyo, 7-3-1 Hongo, Bunkyo-ku, Tokyo 113-8655, Japan; ^3^Department of Neurosurgery, Nippon Medical School, 1-1-5 Sendagi, Bunkyo-ku, Tokyo 113-8602, Japan

## Abstract

The presence of an intracranial aneurysm together with a pituitary adenoma presents tremendous risk of subarachnoid hemorrhage, during transsphenoidal surgery, particularly when the aneurysm lies near the operative field. A left supraclinoid internal carotid artery aneurysm and a clinically nonfunctioning pituitary adenoma coexisted in a 57-year-old woman. Initially, the aneurysm was treated by endovascular coil placement, and then the patient underwent pseudocapsule-based extracapsular resection of the pituitary tumor via a transnasal transsphenoidal endoscopic approach. Pseudocapsule-based extracapsular total resection was safely performed, because of the extirpated risk of rupture of the coil-treated aneurysm. Recently, transsphenoidal pseudocapsule-based extracapsular resection approach for pituitary adenomas provides a more effective and safe alternative compared to the traditional intracapsular one because of its higher tumor removal and remission rates and lower recurrence rate. Compared with conventional subcapsular removal, pseudocapsule-based extracapsular resection has more risks of aneurysmal rupture that is located adjacent to pituitary adenoma. Thus, in a patient having a cerebral aneurysm with the proximity to the operative field, the cerebral aneurysm should be first treated with endovascular coil placement or direct surgical procedure; subsequently, pseudocapsule-based extracapsular resection of the pituitary tumor via a transnasal transsphenoidal endoscopic approach should be performed.

## 1. Introduction

Coexistence of an intracranial aneurysm and a pituitary adenoma has been well documented [1–4]. This association has been reported to range from 3.7% to 7.4%. Subarachnoid hemorrhage due to rupture of an intracranial aneurysm adjacent to pituitary adenoma is a tremendous risk for transsphenoidal surgery. The transsphenoidal pseudocapsule-based extracapsular resection provides a more effective and safe alternative compared to the traditional intracapsular one because of its higher tumor removal and remission rates and lower recurrence rate [5]. In contrast, compared with conventional subcapsular removal, pseudocapsule-based extracapsular resection has more risks of aneurysmal rupture that is located adjacent to pituitary adenoma. In this paper, the clinical management of pituitary adenoma and adjacent cerebral aneurysm is discussed with case presentation.

## 2. Case Presentation

**Figure 1:**
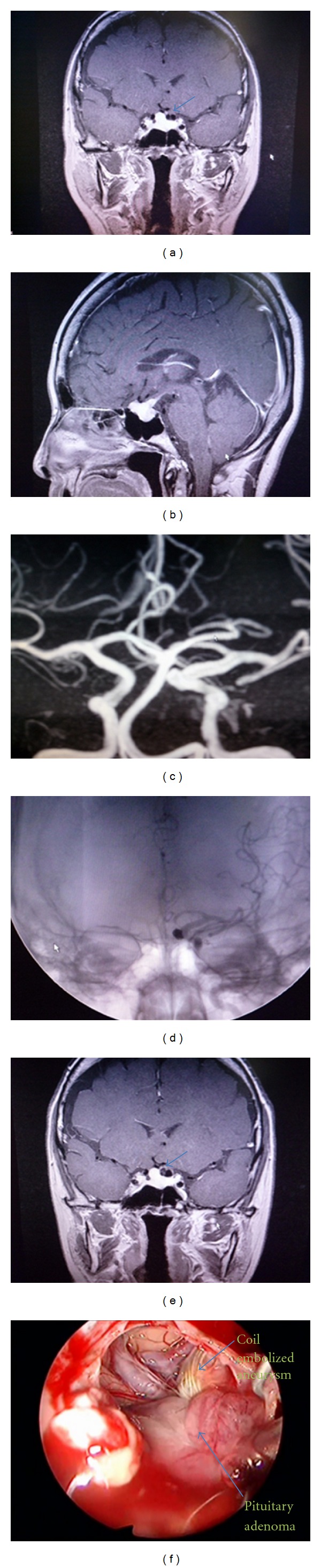
(a) Enhanced-MRI coronal image. (b) Enhanced-MRI sagittal image. An intrasellar mass lesion with suprasellar extension is suggestive of a pituitary adenoma with diameters of 17 × 16 × 11 mm. A flow void mass in the left supraclinoid internal carotid artery raised the suspicion of a cerebral aneurysm ((a) arrow). (c) MR angiography confirms a left supraclinoid internal carotid artery aneurysm (measuring approximately 6.0 mm in diameter). (d) Cerebral angiography. (e) Enhanced-MRI coronal image. The aneurysm is treated by endovascular coil placement ((e) arrow). (f) Intraoperative photograph. Pseudocapsule-based extracapsular resection of the pituitary tumor via a transnasal transsphenoidal endoscopic approach is performed. Coil-embolized aneurysm (arrow) is noted adjacent to pituitary adenoma (arrow).

A 57-year-old woman presented to our hospital with a complaint of dizziness. Neurological examination revealed no abnormalities. Magnetic resonance imaging (MRI) revealed an intrasellar mass lesion with suprasellar extension, suggestive of a pituitary adenoma with diameters of 17 × 16 × 11 mm (Figures 1(a) and 1(b)). On MRI, a flow void mass in the left supraclinoid internal carotid artery raised the suspicion of a cerebral aneurysm (Figure 1(b)), which was confirmed with MR angiography (measuring approximately 6.0 mm in diameter) (Figure 1(c)). Endocrinological studies revealed normal pituitary functions. Initially, the aneurysm was treated by endovascular coil placement (Figures 1(d) and 1(e)). Three years later, the patient underwent pseudocapsule-based extracapsular resection of the pituitary tumor via a transnasal transsphenoidal endoscopic approach (Figure 1(f)). Histological examination confirmed the diagnosis of a clinically nonfunctioning adenoma. Pseudocapsule-based extracapsular total resection of the adenoma was safely performed, because of the extirpated risk of rupture of the coil-treated aneurysm. The patient's postoperative course was uneventful.

## 3. Discussion

Recently, pituitary adenomas are operated via a transnasal transsphenoidal endoscopic approach. Moreover, in the past several years, increasing attention has been paid to the utility of a pseudocapsule in transphenoidal surgery for pituitary adenomas. The transsphenoidal pseudocapsule-based extracapsular resection provides a more effective and safe alternative compared to the traditional intracapsular one because of its higher tumor removal and remission rates and lower recurrence rate [5]. In contrast, compared with conventional subcapsular removal, pseudocapsule-based extracapsular resection has more risks of aneurysmal rupture that is located adjacent to pituitary adenoma.

Cerebral aneurysms can be treated by endovascular or microsurgical techniques. Simultaneous microsurgical treatment of the aneurysm and pituitary adenoma through a frontotemporal [4, 6, 7] or supraorbital keyhole approach [8] was reported. Endovascular embolization of cerebral aneurysm followed by transsphenoidal microsurgery [9–12] or medical therapy [13] was also documented. In the present case, with an aim to prevent the possible risk to the patient from the proximity of the aneurysm to the operative field, the cerebral aneurysm was first treated with endovascular coil placement; subsequently, pseudocapsule-based extracapsular resection of the pituitary tumor via a transnasal transsphenoidal endoscopic approach was performed. Managing strategy of aneurysm treatment first has been usually the safety choice especially for pseudocapsule-based extracapsular resection of the pituitary tumor via a transnasal transsphenoidal endoscopic approach.

## References

[1] Weir B (1992). Pituitary tumors and aneurysms: case report and review of the literature. *Neurosurgery*.

[2] Pant B, Arita K, Kurisu K, Tominaga A, Eguchi K, Uozumi T (1997). Incidence of intracranial aneurysm associated with pituitary adenoma. *Neurosurgical Review*.

[3] Jakubowski J, Kendall B (1978). Coincidental aneurysms with tumours of pituitary origin. *Journal of Neurology Neurosurgery and Psychiatry*.

[4] Wakai S, Fukushima T, Furihata T, Sano K (1979). Association of cerebral aneurysm with pituitary adenoma. *Surgical Neurology*.

[5] Qu X, Yang J, Sun JD (2011). Transsphenoidal pseudocapsule-based extracapsular resection for pituitary adenomas. *Acta Neurochirurgica*.

[6] Fujiwara S, Fujii K, Nishio S, Fukui M (1991). Diagnosis and treatment of pituitary adenoma with adjacent carotid artery aneurysm. *Journal of Neurosurgical Sciences*.

[7] Yang MY, Chen C, Shen CC (2005). Cavernous aneurysm and pituitary adenoma: management of dual intrasellar lesions. *Journal of Clinical Neuroscience*.

[8] Revuelta R, Arriada-Mendicoa N, Ramirez-Alba J, Soto-Hernandez JL (2002). Simultaneous treatment of a pituitary adenoma and an internal carotid artery aneurysm through a supraorbital keyhole approach. *Minimally Invasive Neurosurgery*.

[9] Nishijima Y, Ogawa Y, Sato K, Matsumoto Y, Tominaga T (2010). Cushing’s disease associated with unruptured large internal carotid artery aneurysm. *Neurologia Medico-Chirurgica*.

[10] Sade B, Mohr G, Tampieri D, Rizzo A (2004). Intrasellar aneurysm and a growth hormone-secreting pituitary macroadenoma: case report. *Journal of Neurosurgery*.

[11] Seda L, Cukiert A, Nogueira KC, Huayllas MKP, Liberman B (2008). Intrasellar internal carotid aneurysm coexisting with GH-secreting pituitary adenoma in an acromegalic patient. *Arquivos de Neuro-Psiquiatria*.

[12] Soni A, Silva SR, Allen K, Byrne JV, Cudlip S, Wass JAH (2008). A case of macroprolactinoma encasing an internal carotid artery aneurysm, presenting as pituitary apoplexy. *Pituitary*.

[13] Wang CS, Yeh TC, Wu TC, Yeh CH (2009). Pituitary macroadenoma co-existent with supraclinoid internal carotid artery cerebral aneurysm: a case report and review of the literature. *Cases Journal*.

